# Authors' reply

**DOI:** 10.4103/0019-5413.41866

**Published:** 2008

**Authors:** Allen Li, Jung-Kuei Chen, Kung-Chia Li, Ching-Hsiang Hsieh

**Affiliations:** *Department of Biology, Johns Hopkins University, Baltimore, MD 21218, USA*; 1 *Department of Orthopedic Surgery, Armed Forces Taoyuan General Hospital, Taoyuan, Taiwan, ROC*; 2 *Department of Orthopedic Surgery, Chiayi Yang-Ming Hospital, Chiayi, Taiwan, ROC*; 3 *Institute and Faculty of Physical Therapy, National Yang-Ming University, Taipei, Taiwan, ROC*

Sir,

Thank you very much for your interest in our paper and active comments.^1^

Our postoperative protocol is designed based on the principle of ambulation as early as possible. Therefore, about 80% of our patients with Frankel E and D were ambulatory within 6-8 hours after the operation, the remaining 20%, 8-24 h. We encourage patients to walk as soon and as much as possible. We also believe that most burst fractures without neural deficits will not need any surgery if patients can stay in bed for three months.

For a single burst fracture, we never did long-segment fixation. Therefore, your suggestion to compare the long-segment fixation with TpBA augmentation is not possible at our hospital. Our personal opinion is that long-segment fixation is not really necessary for most burst fractures. Ideally, the single vertebral body problem should be treated over the lesion site only. The fusion involving the intact vertebrae probably is not really needed and should be avoided if possible. What I believed is that the trend to treat burst fracture will be long-segment fixation, then short-segment fixation with anterior support, and finally only vertebra reconstruction itself.

Blood loss and operation time can be controlled in a mature, trained surgon with practice. The figures of blood loss are authantic and seen by more than 200 overseas observers who have observed our procedures critically. You are very welcome to our hospital to observe.

Flexion-extension views are able to check many problems of the spine, including the stability of TpBA and the status of adjacent discs or vertebrae. We just want to make sure everything is right.
